# Catheter‐directed thrombolysis as an emergent intervention for failed vacuum thrombectomy of pulmonary embolism

**DOI:** 10.1002/rcr2.1124

**Published:** 2023-03-23

**Authors:** Syed H. Haq, Jordan Hinegardner‐Hendricks, Cliff Cole, Amanda Laird, Sidra R. Shah, Sandeep M. Patel, William Cole

**Affiliations:** ^1^ Department of Internal Medicine BonSecour Mercy Health—St. Rita's Medical Center Lima Ohio USA; ^2^ Structural Heart & Intervention Center BonSecour Mercy Health—St. Rita's Medical Center Lima Ohio USA; ^3^ Department of Critical Care Medicine BonSecour Mercy Health—St. Rita's Medical Center Lima Ohio USA

**Keywords:** mechanical thrombectomy, pulmonary embolism, Swan–Ganz catheter, thrombosis, tissue plasminogen activator

## Abstract

Acute pulmonary embolism [PE] in the setting of hemodynamic instability and right ventricular strain is a complex presentation and typically is associated with high mortality rates. Prompt recognition and early intervention are critical to the survival of these patient. In such cases, current guidelines recommend use of systemic thrombolytics, along with as needed cardiopulmonary support. If contraindications are present, mechanical thrombectomy is advised. However, guidelines poorly define the next steps in intervention if mechanical thrombectomy were to fail. We present such a scenario and the methods implored to successful remove clot burden. We add to the literature, use of catheter directed thrombolysis at a designated 2 mg/h rate as a form of emergent intervention in failed mechanical thrombectomy.

## Graphical abstract

In this manuscript, we present a novel regimented treatment modality for pulmonary embolism in high‐risk patients who have failed mechanical thrombectomy.

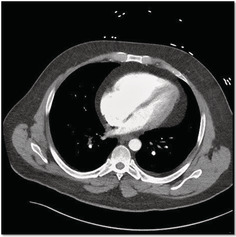

## INTRODUCTION

Pulmonary embolism is a common and potentially life‐threatening presentation of thrombosis seen in the lung, afflicting about one in a thousand Americans annually.^1^ PE can present as shortness of breath, hypoxia, chest pain that is pleuritic in nature and haemoptysis which can rapidly progress to respiratory failure. This makes early diagnosis and treatment critical. Diagnosis is made by computed tomography [CT], which may demonstrate concurrent right heart strain. Echocardiographic evidence suggests an incidence of 30%–70% of normotensive individuals with PE will have right ventricular strain. In such cases, treatment involves the use of systemic tissue plasminogen activator (tPA) or mechanical thrombectomy depending on patient presentation and facility experience.^2^, ^3^ However, guidelines poorly define the next steps in intervention if mechanical thrombectomy were to fail.^4^ This case presents a novel option for treatment after the failure of mechanical thrombectomy or standard anti‐coagulative methods.

## CASE REPORT

**FIGURE 1:**
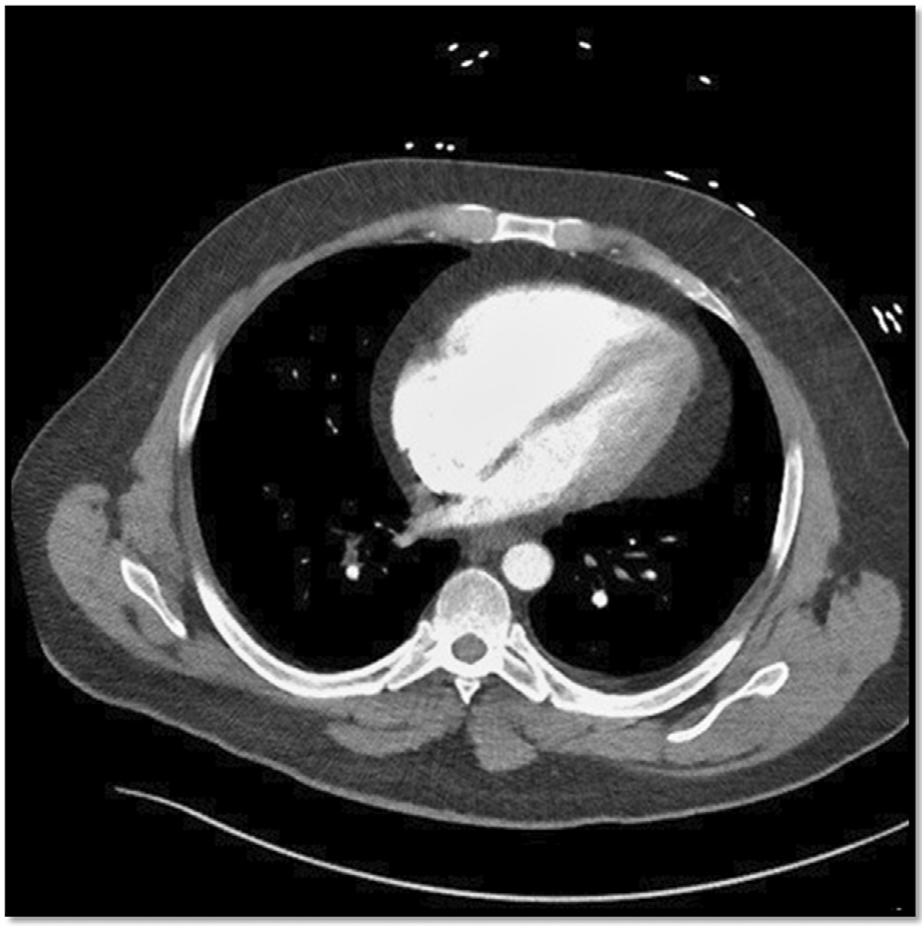
Demonstrates findings for right ventricular strain with an approximate RV:LV >0.9

A 40‐year‐old Caucasian male with a past medical history of obesity presented to the emergency department for complaints of sudden onset shortness of breath, chest pain and hypoxia. Computed tomographic (CT) angiography of the chest revealed a large saddle embolus, pulmonary infarction in the right lung, and an elevated RV/left ventricle, as noted in Figure 1. A STAT echocardiogram revealed preserved left ventricular systolic function, but severe right ventricular dilation and reduced systolic function. The patient subsequently underwent a large bore mechanical thrombectomy. Unfortunately, mechanical thrombectomy failed to resolve the clot burden and right ventricular strain was still noted. Surgical thrombectomy was discussed with cardiothoracic surgery. Due to extreme risk, we explored a percutaneous option.

**FIGURE 2:**
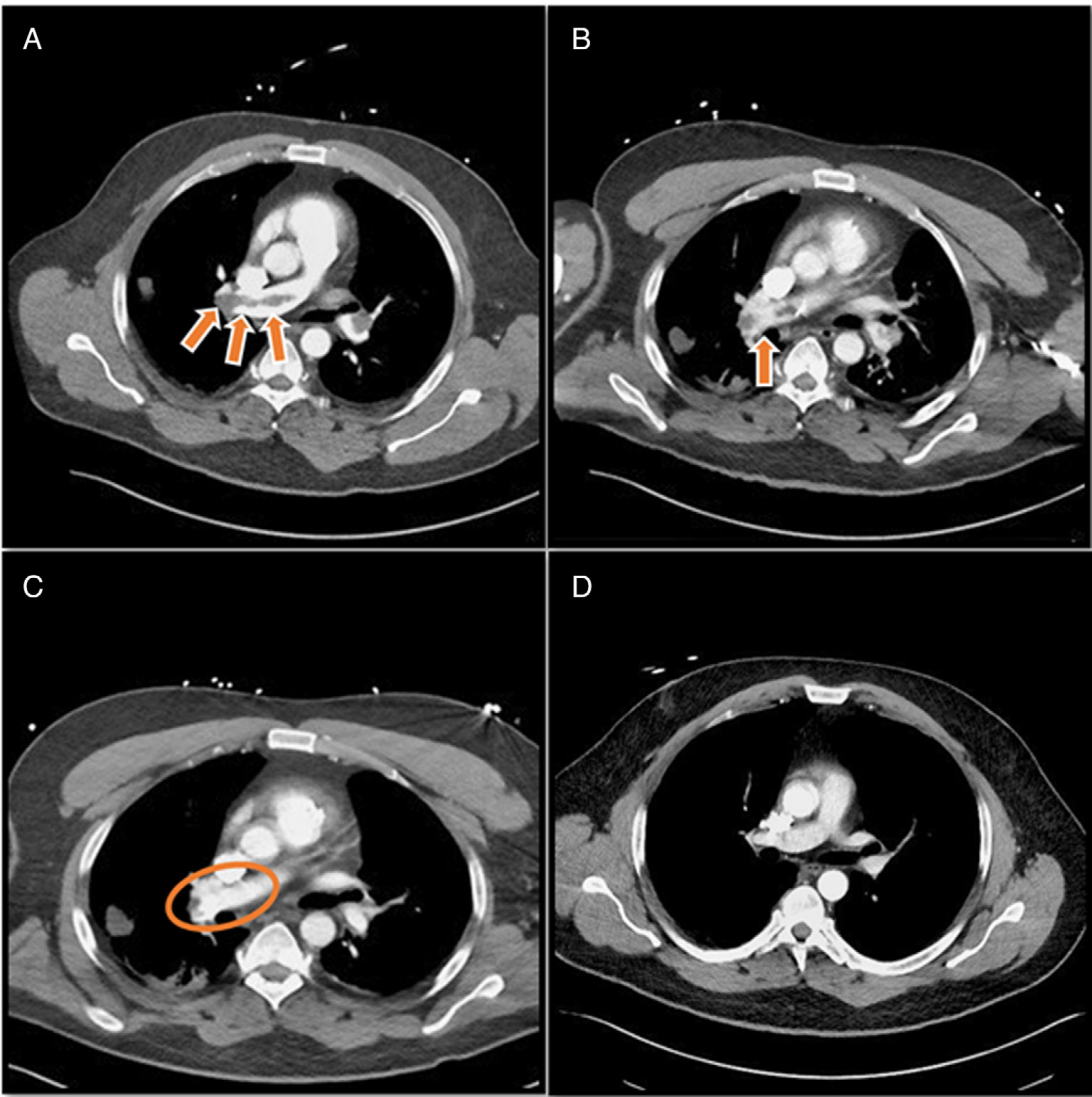
Demonstrates progressive clot reduction by means of catheter directed tPA at 2 mg/h. (A) A extensive thrombus can be visualized at day 0. (B) Illustrates improvement in cloth burden at day 1 of tPA. (C) There is a significant noticeable resolution at day 3 of tPA. (D) Follow up CT scan at 6 months demonstrating no clot burden

A subclavian Swan–Ganz catheter (SGC) was placed, through which alteplase was administered at a rate of 2 mg/h via a distal port. Infusion would be continuous until significant symptomatic improvement with decreased clot burden on imaging, fibrinogen levels fall below 150, or a complication were to arise. Baseline fibrinogen was established at 375 and monitored every 4 hours. At 24 h of continuous administration of alteplase, fibrinogen levels dropped to 255. A follow‐up CT demonstrated a reduction in clot burden with contrast penetrating into the clot, as seen in Figure 2B. After 3 days of alteplase administration, there was noticeable improvement in clinical presentation and symptoms. A subsequent CT scan demonstrated substantial clot regression, as seen in Figure 2C. Alteplase was discontinued and 2 days later the patient was discharged to home on apixaban. A final repeat CT scan at 6 months shows no clot burden, as seen in Figure 2D.

## DISCUSSION

Currently, few methods have been elucidated for intervention in the care of a patient suffering from a pulmonary embolism after a failed mechanical thrombectomy. This case presents one potential method of high‐level treatment of severe pulmonary embolism when mechanical thrombectomy is unsuccessful.

Catheter‐directed therapy has been utilized as either first line treatment, salvage therapy after failure of treatments such as thrombolysis or as bridge therapy to stabilize patients until other treatments are sought.^3^ However, this case is novel based upon three characteristics including catheter‐directed therapy after failed mechanical thrombectomy, dosing schedule, and length of treatment. A thorough literature review returned no specific cases utilizing catheter‐directed therapy with SGC as salvage post failed mechanical thrombectomy. Advantages of SGC utilization include significantly decreased cost of catheter, expeditious placement, and the ability to place in the ICU. The use of the SGC makes it a ubiquitous method of tPA delivery that can be employed by many practitioners.

The literature search also found no dosing schedule for tPA at 2 mg/h with catheter‐directed therapy. Most protocols utilize tPA at a maximum of 1 mg/h. Efficacy and safety of higher tPA administration are supported where 2 mg/h tPA is recurrently and safely administered intra‐arterially to treat cerebral venous sinus thrombosis.^5^ Finally, the length of therapy at 3 days duration appears to be novel regarding catheter‐directed thrombolysis as most protocols utilize tPA for a maximum of 36 h. The decision to stop therapy, in this case, was based on symptomatic improvement and radiograph clot regression.

This case outlines the successful implementation of a novel protocol for treating pulmonary emboli with right ventricular strain when mechanical thrombectomy fails. The advantages of our approach hinge on the universality of SGC use, low‐cost alternative to current catheter‐based approaches, and the availability of tPA at almost all institutions that manage critically ill patients, making this a practical, viable, and rapidly implementable protocol for such patients.

## AUTHOR CONTRIBUTIONS

Primary authors; Syed Haq, Jordan Hinegardner‐Hendricks and Cliff Cole. Data gathering and research assistance; William Cole and Amanda Laird. Editors; Syed H. Haq, Sidra R. Shah and Sandeep M Patel.

## CONFLICT OF INTEREST STATEMENT

None declared.

## ETHICS STATEMENT

The authors declare that appropriate written informed consent was obtained for the publication of this manuscript and accompanying images.

## Data Availability

Data sharing not applicable to this article as no datasets were generated or analysed during the current study
